# Causes and Consequences of Variable Tumor Cell Metabolism on Heritable Modifications and Tumor Evolution

**DOI:** 10.3389/fonc.2020.00373

**Published:** 2020-03-27

**Authors:** Bryce Ordway, Pawel Swietach, Robert J. Gillies, Mehdi Damaghi

**Affiliations:** ^1^Department of Cancer Physiology, H Lee Moffitt Cancer Center and Research Institute, Tampa, FL, United States; ^2^Department of Physiology, Anatomy and Genetics, University of Oxford, Oxford, United Kingdom; ^3^Department of Oncologic Sciences, Morsani College of Medicine, University of South Florida, Tampa, FL, United States

**Keywords:** tumor evolution, acidosis and oxidative stress, nutrient sensing and signaling, tumor micoenvironment, epigenetic regulation

## Abstract

When cancer research advanced into the post-genomic era, it was widely anticipated that the sought-after cure will be delivered promptly. Instead, it became apparent that an understanding of cancer genomics, alone, is unable to translate the wealth of information into successful cures. While gene sequencing has significantly improved our understanding of the natural history of cancer and identified candidates for therapeutic targets, it cannot predict the impact of the biological response to therapies. Hence, patients with a common mutational profile may respond differently to the same therapy, due in part to different microenvironments impacting on gene regulation. This complexity arises from a feedback circuit involving epigenetic modifications made to genes by the metabolic byproducts of cancer cells. New insights into epigenetic mechanisms, activated early in the process of carcinogenesis, have been able to describe phenotypes which cannot be inferred from mutational analyses *per se*. Epigenetic changes can propagate throughout a tumor via heritable modifications that have long-lasting consequences on ensuing phenotypes. Such heritable epigenetic changes can be evoked profoundly by cancer cell metabolites, which then exercise a broad remit of actions across all stages of carcinogenesis, culminating with a meaningful impact on the tumor's response to therapy. This review outlines some of the cross-talk between heritable epigenetic changes and tumor cell metabolism, and the consequences of such changes on tumor progression.

## Molecular Mechanisms of Microenvironmental Sensing

Cancer evolution operates through selection, which requires a degree of phenotypic diversity to present a range of possible responses to microenvironmental selection forces, some of which confer selective advantage (1, 2). Tumors can be described as complete ecosystems, containing cancer cells, stromal cells, vasculature, extracellular matrix, and the chemical milieu consisting of variables such as pH and oxygen tension (3–5). During tumorigenesis—and similarly in response to therapy—the tumor ecosystem shows considerable plasticity because cancer cells shape their microenvironment, to which subsequent generations of them must adapt to thrive, and these adaptations, in turn, fine-tune the microenvironment (6). During the various stages of tumor progression, cells can be exposed to highly variable chemical stimulations, largely attributed to variable blood perfusion; for example, oxygen deprivation (hypoxia), nutrient deprivation, metabolic end-product build-up, and increased acidity. Overall, these stimuli would be considered, by normal standards, to be survivable to most cells but exerts some cost on cells fitness, and it is therefore axiomatic that cancer cells must adapt to these conditions if they are to thrive. Although the stimuli are survivable, they still impose stress on the cells which changes their fitness, requiring acquisition of a novel homeostatic balance that costs more energy for cells and can be lethal for cells in competition with other cells.

Hypoxia is one of the main environmental factors a cancer cell must face in order to survive, thrive, and progress. Hypoxia imposes a metabolic stress on the cell, hindering its ability to carry out aerobic respiration. Therefore, the cell must be able to adapt to a hypoxic environment in order to survive. The cellular response to hypoxia is robust, and exerts most of its force via the transcription factors HIF-1α and HIF-2α. Another HIF family protein, HIF-3α, functions to repress the responses directed by HIF-1α and HIF-2α. All three of these proteins carry out their function via dimerization with constitutively expressed HIFβ proteins in the nucleus which allows them to directly modulate transcription of proteins involved in the hypoxia response (7, 8), or in the case of HIF-3α repress the transcriptional response. HIF-1α is a constitutively expressed protein, whose activity is regulated by the hydroxylation of conserved proline residues. In microenvironments of high oxygen tension (>5%), the proline residues are hydroxylated, tagging the protein for degradation by E3 ubiquitin ligases (9). When oxygen concentrations are below the tolerable threshold for a given cell type, HIF-1α is not degraded, and increases in concentration to allow HIF-1α to induce transcription of its client genes (10).

Nutrient deprivation is another major stressor within the tumor microenvironment. When the cell experiences a critical reduction in a particular nutrient, it must swiftly adjust in order to maintain productivity in the terms of metabolism, proliferation, migration, or other processes essential to evolutionary success and survival. Two main nutrient sensing proteins implicated in cancer are AMP-activated protein kinase (AMPK) and the mammalian target of rapamycin (mTOR) (11, 12). These proteins are capable of sensing the current energy status of the cell and nutrient availability, respectively.

The metabolic pathway directed by AMPK is highly context-specific; depending on the nutrient status of the cell. AMPK is responsible for the cellular response to glucose deprivation and acts as a metabolic switch from a highly glycolytic state to an oxidative state depending on the availability of glucose. This is particularly important in the context of cancer and the highly plastic nature of cancer metabolism. AMPK is activated by 5′-AMP, which indicates that the cell is not regenerating ATP at a fast-enough rate to meet demand. This induces the uptake of glucose and the induction of glycolysis to replenish the cellular ATP (11). The induction of glycolysis via-a-vis respiration is likely due to the promptness with which glycolytic activation can occur (13).

mTOR is present in the form of two different complexes, mTORC1 and mTORC2. These two complexes participate in associated, but distinct signaling pathways in nutrient sensing. mTORC1 becomes activated in response to various growth factors and amino acids that promote cell growth and proliferation. When inactive, mTORC1 represses growth and induces an autophagic response. mTORC2 is a sensor of glucose but also plays a role in amino acid signaling. mTORC2 is activated by acetyl-coenzyme A (Ac-CoA), which is produced in the cytoplasm via citrate lyase, when glycolytic flux is abundant. The result of mTORC2 activation is increased cell proliferation, in response to the increased glucose metabolism. mTORC2 has also been implicated in amino acid sensing by having the ability to suppress the function of the glutamine-cysteine transporter, system Xc transporter-related protein (12).

Aberrant perfusion in the tumor microenvironment allows a significant build-up of metabolites in the tumor interstitial fluid. The main metabolite that is commonly accumulated in the tumor interstitial fluid is lactic acid, which is associated with a decrease in pH. A decrease in extracellular and intracellular pH can dramatically modulate the activities of enzymes, some of which are more sensitive than others, depending on how significant the change in pH is and the isoelectric point of the enzymes optimal activity (14). These alterations in enzyme activity are pleiotropic and leads to metabolic reprogramming. Lactate in the tumor microenvironment is a by-product of increased glucose fermentation, which occurs even in the presence of oxygen, known as aerobic glycolysis or the Warburg Effect. Once produced, lactate is shuttled out of the cell, stoichiometrically with a proton, by monocarboxylate transporters (MCTs) 1–4. Sensing of extracellular pH is accomplished through a variety of plasma membrane associated proteins including two major classes of acid-sensing receptors: (i) G-protein coupled receptors (GPCR) such as Ovarian cancer G protein-coupled receptor 1 (OGR1), G-protein coupled receptor 4 (GPR4), T-cell death-associated gene 8 (TDAG8), and ii) Acid-sensitive ion channels (ASICs) which include 7 proteins from 4 genes: 1a/b,2a/b,3,4,5, and Ca^2+^ channel that includes transient receptor protein channel vanilloid subfamily 1 and 2 (TRPV1 and TRPV2) (15). Lactate can also be sensed and regulate cellular functions by activating the G protein-coupled receptors HCA_1_/GPR81, HCA_2_/GPR109A, and HCA_3_/GPR109B. These hydroxy-carboxylic acids (HCA) receptors control physiological homeostasis under changing metabolic and dietary conditions (16).

**Figure 1:**
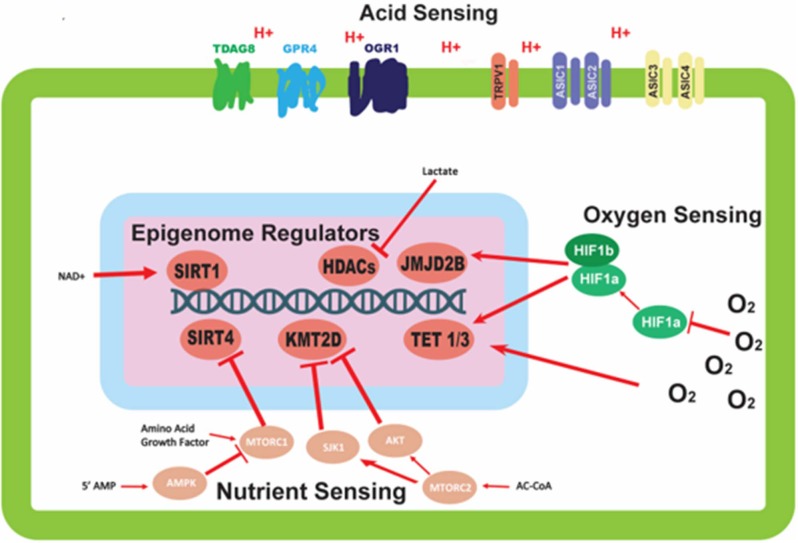
Mechanisms of environmental sensing and their effects on epigenetic modifiers. This figure depicts the various avenues by which cells are able to detect perturbations in the environment, and the downstream effects of this sensing on epigenetic modifiers. Oxygen sensing mechanisms trigger dimerization of HIF-1α and HIF-1β proteins to affect epigenetic modifiers; oxygen also direct modulates activity of epigenetic modifiers. Nutrient sensing can initiate a signaling cascade, largely mediated by the mTORC complexes and AMPK protein, with the ability to alter the activity of epigenetic modifying proteins. Acid sensing proteins located on the outer membrane of the cell are able to sense the extracellular pH, but have no clear mechanism for altering the activity of epigenetic modifiers.

Cancer cells commonly overexpress many of the aforementioned acid sensors, and this can be correlated to tumor progression and poor outcome (17, 18). Therefore, investigating these sensors as a factor in malignancy may identify relevant prognostic biomarkers or may reveal new therapeutic vulnerabilities. The sensors can be connected to pathways to activate transcription factors or overexpress other genes and proteins (Figure 1). However, considering the ever-changing state of the microenvironment, we propose that epigenetic regulation may be a more effective factor in stabilization of emerging phenotypes in cancer cells. Adaptation to an acid-microenvironment has been shown to alter cell state by pushing cells into a partial EMT phenotype (19); this may be a manifestiation of these acid sensors inducing a stable epigenetic change.

## Heritable Epigenetic Modifications Acquired Through Microenvironmental Sensing

One mechanism enabling cancer cells to adapt is through changes to gene expression via epigenetic regulation. Some modalities of epigenetic regulation are transient (e.g., histone acetylation) and are imposed to help cancer cells survive acute disruptions in their microenvironmental homeostasis. In contrast, other epigenetic mechanisms are more persistent (e.g., DNA methylation) and have the ability to be passed down through generations to endow further generations with memory on how to survive in the tumoral microenvironment.

The term epigenetics was first created by CH Waddington who described it as “the causal interactions between genes and their products, which bring the phenotype into being.” While a commonly agreed upon definition is hard to find today, the term epigenetics in the modern era is commonly described as a permanent change in the way genes are expressed. Types of epigenetic regulation include histone modifications, DNA Methylation, and non-coding RNA (20), which can all impact one another to create a complex regulatory dynamic. A major question is: “How do the external factors of the tumoral microenvironment play into altering this complex dynamic”?

It is commonly known that the epigenetic signatures of cancer cells are different compared to their untransformed counterparts (21–25). Many of these epigenetic alterations exert their function by altering the metabolism of cancer cells (26), and are acquired by signaling cascades initiated by sensing of the extracellular environment. Some of the signaling cascades that can lead to changes of epigenetic signatures were implied in the previous section and the specific alterations they are involved in will be discussed herein.

In many solid tumors, intra-ductal hyperplasia leads to significant alterations in the physical microenvironment, especially in peri-luminal cells that are far (>160 microns) from their blood supplies. Importantly, the diffusion distance of oxygen in tissues is 100–200 microns (27), meaning that the periluminal cells of DCIS can be profoundly hypoxic. The depth and duration of hypoxia is dependent on the blood flow of the surrounding stroma. Hypoxia eventually selects for metabolic reprogramming, leading to acidosis, as well as exacerbating nutrient deprivation. Over many years in this environment, these forces (hypoxia, acidity, nutrient deprivation) select for cells with more highly adaptable, aggressive, de-differentiated phenotypes (6, 28). The resulting acidosis leads to genome instability, which could increase the rate of cancer evolution (29). The source of cytoplasmic (and nuclear) acidosis is lactate accumulation as a byproduct of glycolysis. Lactate has been shown to have a variety of effects on the epigenetic mechanisms of the cell, some of which are confounding. Direct inhibition of histone deacetylases (HDACs) by lactate has been shown in separate studies (30, 31); while others have reported multiple times on the increase in activity of the Sirtuin family of histone deacetylases upon exposure of cells to a chronically acidic extracellular environment (32, 33). These examples represent combined sensing and epigenetic effector mechanisms that act directly on altering the epigenetic status of the cell.

The oxygen sensing protein HIF-1α can regulate epigenetics in a variety of ways. Upon activation, HIF-1α leads to downstream signaling cascades important for the survival of cells in low oxygen environments. The ultimate effect of some of these signaling cascades is the epigenetic alteration of gene regulation (Figure 1). Two epigenetic mechanisms influenced by HIF-1α are histone methylation and DNA methylation (Figure 1). Unlike other epigenetic mechanisms, histone methylation can act in both activating and repressing fashions depending on the specific location of the covalent modification. The histone demethylase JMJD2B is activated by HIF-1α (Figure 1), and is specifically targeted to H3K9me2/3 to demethylate the mark to a monomethylated state (34, 35). The expression of ten-eleven translocation proteins 1/3 (TET1/3) is also upregulated upon stabilization of HIF-1α. TET1/3 are 5-methylcytosine (5mC) oxidases, which convert 5mC into either 5-hydroxymethylcytosine (5hmC), 5-formylcytosine (5fC), or 5-carboxylcytosine (5caC) via sequential reactions (36). The result of these reactions is the deactivation of the methylation mark and the subsequent reactivation of the sequence being repressed by the methylation. This has been validated in neuroblastoma where it was shown that HIF-1α can induce HIF-1α/hypoxia specific DNA methylation signatures (37). The fact that HIF-1α activates DNA methylation supports our hypothesis of inheritable epigenetic changes to next generation that can be tracked in cancer cells. Contrary to the upregulation of TET by hypoxia-induced transcriptional programs, TET proteins have been shown to have their activity reduced directly by low oxygen availability in a tumor setting. TET activity is lost *in vitro* when exposed to hypoxic conditions, possibly via hypermethylation of tumor suppressor promoters in hypoxic regions of tumor samples (38). The opposing forces of the transcriptional and functional regulation of TET proteins may demonstrate a physiological feedback system for regulating the epigenetic response to oxygen deprivation in order to attenuate the response (Figure 1).

**Figure 2:**
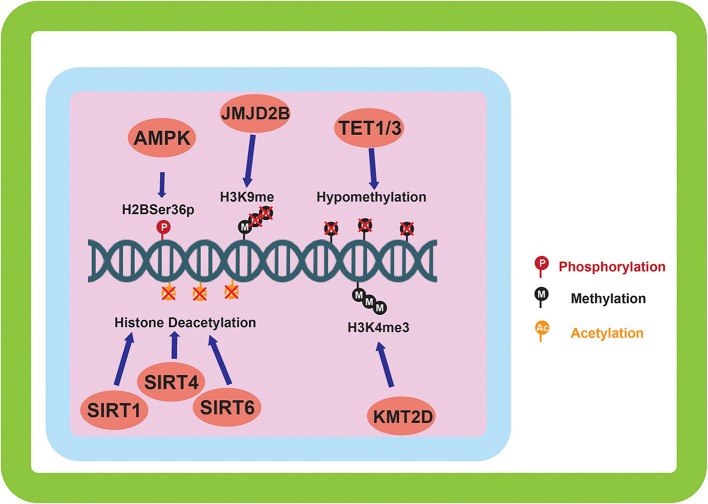
Epigenetic alterations mediated by environmentally induced epigenetic modifiers. The epigenetic modifiers altered by the sensing of the cellular environment go on to carry out a variety of modifications to the epigenome. DNA methylation, and histone methylation and acetylation are the main downstream targets of the environmental sensing. Phosphorylation of histones by AMPK is also carried out in response to environmental sensing.

The sensing of nutrients by a cell is vitally important to its survival and can have long term effects on the downstream lineage of that cell via epigenetic modifications. Having this feed forward system of epigenetic regulation directed by nutrient signaling allows for increased fitness for subsequent generations. As mentioned previously, cellular nutrient sensing is mainly achieved through 3 essential proteins and protein complexes: AMPK, mTORC1, and mTORC2 (Figure 1). AMPK is activated in response to cellular metabolic stress, and modulates gene transcription epigenetically in order to respond to this stress. Unlike the mTORC1/2 complexes, AMPK is able to modulate transcription directly by phosphorylation of Ser^36^ on histone H2B (39). This phosphorylation mark directly promotes the transcription of response genes needed to handle metabolic stress. Other papers report the direct phosphorylation of Ser^36^ on H2B by S6K1 (40), which is also a player in the LKB1-AMPK-mTORC1 signaling axis, with S6K1 being phosphorylated by mTORC1. mTORC1 is another player in the epigenetic response to nutrient sensing. As mentioned previously, mTORC1 is capable of sensing various growth factors and amino acids. A downstream target of mTORC1 nutrient sensing is SIRT4, which is repressed in response to mTORC1 activation (41). SIRT4 is a lysine deacylase (Figure 2) (42), that has the ability to inhibit glutamine metabolism by inhibiting glutamate dehydrogenase (GDH). This inhibition of SIRT4 is achieved at the transcriptional level by mTORC1 stabilizing the CREB2-βTrCp complex, preventing CREB2 from activating transcription of SIRT4 (41). mTORC2 exerts its epigenetic function by activation of the AKT and SGK1 proteins. The effect of these proteins on epigenetic regulation is the inhibition of KMT2D, which is a histone methyltransferase specifically targeting H3K4 (Figure 2) (43). Inhibition of KMT2D has been shown to have anti-tumor effects in some cancers by not allowing the FOXA1-PBX-ER complex to access the DNA for transcription (44).

While the aforementioned mechanisms involve the sensing of nutrients to transduce downstream epigenetic changes, alterations in acetate level can directly influence the epigenetic status of the cell. Free Acetyl-CoA in the cell nucleus is produced by Acetyl-CoA synthetase (ACSS2), which catalyzes the conversion from Acetate, and by ATP-citrate lyase (ACLY) which catalyzes the conversion from citrate. The levels of free Acetyl-CoA directly influence the global acetylation status of histones (45), and henceforth have the ability to regulate epigenetics without the direct manipulation of an enzyme intermediate. While there is no direct sensing mechanism, this level of regulation could be seen as a sensor of the glycolytic state of the cell considering it has been shown that decreasing the amount of glucose available to a cell reduces the Acetyl-CoA abundance and lowers global histone acetylation (46).

A recently described mechanism of both environmental sensing and epigenetic modification is that of histone lactylation. In 2019, Zhang et al. described for the first time the modification of histones by lactate (47). As is commonly known, lactate is built-up as a byproduct of glycolysis. This epigenetic modification may provide a direct mechanism for the regulation of gene expression in response to fermentative glycolytic activity of the cell.

## Effect of Heritable Epigenetic Modifications on Tumor Metabolism

Many epigenetic alterations that are acquired throughout tumor progression alter the metabolism of the tumor's cellular population. While the previous section covered specific epigenetic alterations that occur in response to the metabolic microenvironment, this section will describe the role of epigenetic modifications in altering cancer cell metabolism.

Lactate itself has the ability to alter the activity of epigenetic modifier proteins. The inhibition of HDAC's by lactate demonstrated in previous studies (30, 31) has yet to be phenotypically implicated in the alteration of metabolic processes; yet it is likely this lactate-mediated mechanism will play a role in altering metabolism. The activation of SIRT1 by extracellular acidosis, which is a consequence of acid-inhibition of glycolysis and the subsequent build-up of NAD^+^, has been shown to alter cellular metabolism through histone deacetylation leading to increased transcription of HIF-2α. This SIRT1/HIF-2α axis promotes the oxidative metabolism of glutamine, and suppresses the effects of HIF-1α, inhibiting hypoxia mediated induction of glycolysis (32). Corbet et al. in a later study showed that SIRT1 as well as SIRT6 are essential for histone deacetylation and the induction of fatty acid metabolism when cells are chronically exposed to an acidic extracellular environment (33). From a cell survival standpoint, this switch to other methods of energy metabolism when lactate has accumulated is intuitive, and has relevancy in the context of cancer progression that is discussed later.

In response to hypoxia, HIF-1α activation leads to the induction of JMJD2B activity. JMJD2B has been shown to be upregulated in ER-positive breast cancer (48) and bladder cancer (49), and its upregulation has been directly linked to induction by HIF-1α in colorectal cancer (50) and gastric cancer (51). This activation of JMJD2B directly drives the demethylation of H3K9me2/3 to its monomethylated state (Figure 2). JMJD2B has been shown to play a role in altering the expression of many cancer associated genes including cyclin-dependent kinase 6 (CDK6) (49), and carbonic anhydrase 9 (CA9) (50), which can directly affect the transmembrane pH gradient. Also induced by hypoxia and HIF-1α activation are the expression of TET proteins 1 and 3 (36). As mentioned previously, TET proteins are 5mC oxidases that allow for the expression of genes repressed by DNA methylation. Induction of TET in neuroblastoma has been shown to increase transcription of hypoxia response gene (52), and TET1 has been shown to be overexpressed in triple negative breast cancer (TNBC) where it is associated with hypomethylation (Figure 2) (53). Hypomethylation increases the expression of associated genes such as Hexokinase II (HK2) in liver cancer (54) and glioblastoma multiforme (55). Hexokinase II catalyzes the conversion of glucose to glucose-6-phosphate, an essential step in glycolysis. While CA9 and HK2 are both direct transcriptional targets of the HIF-1α mediated hypoxic response, this epigenetic regulation is important because it imposes the upregulation of these proteins over longer time scales (Table 1) and allows for the maintenance of the metabolic phenotype independently of oxygen status.

**Table 1:** Represenation of the types of epigenetic modifications that can be induced by environmental sensing, and the specific modifications made.

**Types of epigenetic modifications**	**Half-Life (h)**	**Readers**	**Writers**	**Erasers**	**References**
DNA Methylation	Indefinite	MeCP2, MBD1/2/3/4, UHRF1/2, Kaiso, ZBTB4/38	Dnmt1/3a/3b	TET1-3	(70–73)
Histone Acetylation	0.88–1.45	Bromodomain containing enzymes	HATs	HDACs, SIRT1-7	(74–76)
Histone Methylation	5	Proteins containing PHD, chromo, tudor, PWWP, WD40, BAH, ADD, ankyrin repeat, MBT, and zn-CW domains.	Histone Methyltransferases	KDM1 Family proteins, and JMJC Domain-Containing Demthylases	(77–81)
H3K9me3	221	PRC Complex	SUV39H1, SETDB1/2	JMJD2/KDM4, LSD1	(82, 83)
H3K27me3	158	BAHD1	EZH2	JMJD3	(40, 82, 84, 85)
H3K9me2	28.5	PRC Complex	G9a/GLP, PRDM Family	JMJD2/KDM4	(82, 83)
Histone Lactylation	Unknown	Unknown	Lactate Accumulation	Unknown	(47)
**Histone Phosphorylation**					
H2BSer^36^	Unknown	Unknown	AMPK, S6K1	Unknown	(39, 40)

*Informtion including the half-life of the modification, and how the modifications are written, erased, and read is included. This demonstrates the wide variety and complexity of epigenetic modifications that can be made in response to sensing, and the time-scales at which their effects are applicable*.

Epigenetic marks induced by nutrient sensing proteins and complexes have the ability to greatly alter cellular metabolism, making a useful feed-forward mechanism for acclimation and adaptation to the current metabolic microenvironment. The phosphorylation of Ser^36^ on Histone 2B is a significant epigenetic mark made by two proteins involved in nutrient sensing: AMPK and S6K1 (39, 40). It has been shown that phosphorylation of Ser^36^ on Histone 2B is significantly increased upon treatment of cells with 2-Deoxy Glucose (39), which mimics a glucose deprived environment. The resulting effect on the cellular transcription from phosphorylation of Ser^36^ on Histone 2B by AMPK is the recruitment of EZH2 (40). EZH2 is a histone methyltransferase that trimethylates Lysine 27 on histone 3 when recruited. It has been shown in Drosophila that trimethylation of Lysine 27 on histone 3 reduces the glycolytic tendencies of the cell (56). Considering the presence of this mark in glucose-deprived cellular states, it would intuitively make sense that the presence of this mark would decrease the glycolytic capacity of the cell.

The suppression of SIRT4 by mTORC1 has profound effects on the metabolism of cancer cells, specifically inhibiting glutamine metabolism through inhibition of GDH. In colorectal cancer, decreased SIRT4 expression has been correlated with progression and increased invasive potential of cancer cells (57), and in both colorectal and gastric cancers lower SIRT4 expression is associated with poor prognosis (57, 58). All in all, this leads to the conclusion that when in the presence of sufficient amino acids and growth factors, the activation of mTORC1 will lead to the inhibition of SIRT4 and the subsequent reactivation of glutamine metabolism which can promote tumor growth (Figure 2).

mTORC2 has the ability to modulate activity of KMT2D. KMT2D is inhibited downstream during mTORC2 activation, which in response, inhibits the access of the FOXA1-PBX1-ER complex from binding the DNA. Prevention of this complex from binding the DNA has been shown to reduce the expression of key proteins including: GREB1, SERPINA1, cFOS, and MYC (59). Of these proteins, MYC has been shown to have the most substantial effects on reprogramming cancer metabolism in a type-specific manner. A comprehensive list of metabolic alterations in specific cancer types driven by MYC has recently been reviewed (60). The effect MYC has on glycolysis is highly variable depending on the cancer type, with a MYC-associated increase in non-small cell lung cancer and hepatocellular carcinoma, and a MYC associated decrease in renal cell carcinoma and prostatic intraepithelial neoplasia. MYC's effect on glutaminolysis was cohesive in the various cancer types, with an associated increase demonstrated in hepatocellular carcinoma, pancreatic ductal adenocarcinoma, and renal cell carcinoma.

In addition to the previously mentioned alterations in gene expression caused by stimulus-induced epigenetic modifications, epigenetic upregulation of MCT4 via hypomethylation of the SLC16A3 promoter has been shown in renal cancers (61). Although no specific mechanism can be tied to this alteration, this increase in MCT4 expression will have profound consequences on the long-term progression and evolution of the tumor.

## Consequences of Epigenetically Altered Metabolism on Tumor Progression

**Figure 3:**
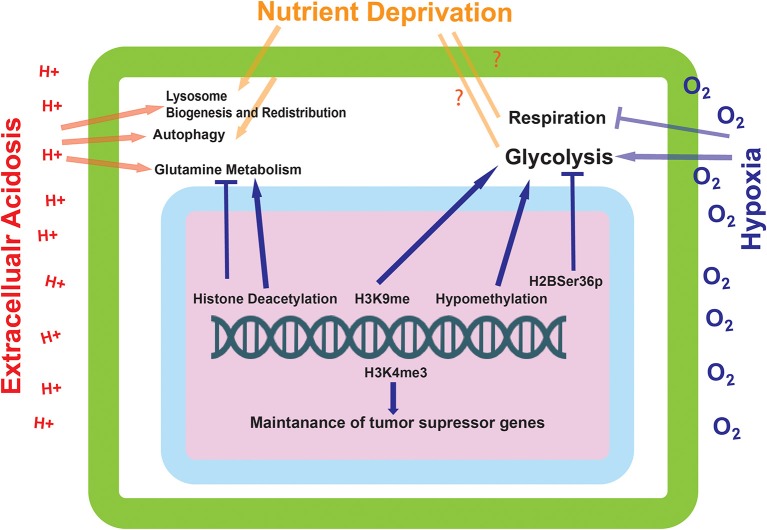
The effect of environmentally induced epigenetic modifications on tumor cell expression and metabolism. The epigenetic modifications made by the proteins influenced by the environmental sensing mechanisms go on to alter the metabolic state of the cell. These modifications can alter the cells ability to metabolize glutamine and carry out glycolysis, as well as influence the transcriptional status of tumor suppressor genes.

As described previously, a resulting consequence of oxygen deprivation and stabilization of HIF-1α is the induction of TET1/3 and hypomethylation of the genome. Hypomethylation has been shown to upregulate the expression of Hexokinase 2 (54, 55), which is associated with an increase in glycolysis (Figure 3). Increased glycolysis will increase acidosis in the tumor microenvironment that can induce extracellular matrix remodeling (15). Thus, increased glycolysis and its sequelae are barriers that cancer cells must overcome in order to meet the energy demands of rapid proliferation and to survive and thrive in a more hostile environment. Altered glycolysis can also lead to Warburg phenotype leading to even more acidic microenvironment and more altered genome and epigenome alteration (28, 62).

Despite the differing sensing mechanisms of the mTORC1/mTORC2 complexes and SIRT1, all mechanisms converge on a single metabolic alteration caused by epigenetic modification: the increase of glutamine metabolism. This may pose the opportunity to target glutamine metabolism as a cancer therapeutic, an idea that has drawn enough attention to warrant various reviews on that subject alone (63, 64). Although this may seem like a rational therapeutic target, cautionary narratives have been proposed as to the possible outcome of creating a resistant cellular population with a heightened metabolic capacity (65). While glutamine metabolism is non-essential in a normally proliferating cell, under periods of rapid proliferation, like tumor growth, glutaminolysis is an essential process (66). This phenotype is selected for due to the high demand for metabolic building blocks produced from the TCA cycle. In the TCA cycle alpha-ketoglutarate can be carboxylated to citrate, which, if in abundance, is translocated to the cytoplasm where it is used for fatty acid synthesis. The end product of glutaminolysis is alpha-ketoglutarate, which is shunted into the TCA cycle to accelerate the process (67). Supporting the TCA cycle with the necessary building blocks will give more chance to glucose to be turned into lactate in glycolysis and augment a Warburg phenotype. Glutamine-fed TCA cycle can also give more freedom to cancer cells to use glycolysis for their fluctuating ATP demand as a quick local source of energy for cancer cells (13).

## Discussion

Genomic data describing tumors and cancer cell populations is valuable information for studying and classifying a cancers phenotypic characteristics. Although this information is valuable, it does not tell the entire story when it comes to cancer initiation and progression. Recent studies have demonstrated the presence of single and multiple driver mutations in significant proportions of cells comprising healthy tissue (68, 69). This poses the question as to how cancer arises, as the once thought sufficient accumulation of driver mutations has been discounted. While it is no doubt these mutations are necessary and play a significant role in cancer progression, it is clear there is more to the story.

Metabolic cross-talk and feedback is essential for the survival of any cell in a highly variable environment. There are many ways cells can sense perturbations in their microenvironment, including nutrient, and energy demands. These sensing mechanisms can transduce signals that lead to the alteration of the cell's epigenome. The epigenetic alterations driven by the alterations to the cellular environment and metabolic state can go on to influence the metabolism of the cell in a feed-forward mechanism. These changes made can have long lasting and heritable effects and go on to influence the progression of the tumor.

Nutrient sensing by cells is essential for the survival and acclimation to an unstable environment (Figure 3). Permanent changes induced by a given environment may be beneficial to a cells lineage, in that they are pre-programmed to deal with the environments endured by their predecessors. In cancer, cells are dynamically exposed to a variety of environmental conditions that would be extremely difficult to survive without epigenetic acclimation handed down from predecessors. Founding cancer populations may have a difficult time surviving the variety of harsh environmental factors, but genetic reprogramming facilitated by epigenetic change would allow the daughter populations to have an increased fitness.

The hyper-glycolytic state of cancer cells is a hallmark of their progression and aggressive state (28). At present, a conclusive mechanism as to the induction of this glycolytic state has yet to be achieved. Described in this paper are various mechanisms in which a cell is able to sense its' current environmental and energetic state of being, some of which can lead to long-lasting changes in the metabolic programming of a cell. Many of these semi-permanent changes converge on the regulation of cellular energetics, in particular, glycolysis. It is therefore conceivable that the mechanism for the induction of a glycolytic state in cancer cells may not be a proposed “switch” or genetic mutation, but instead the accumulation of various epigenetic alterations that permanently reprogram the cellular population (Figure 3). It is possible that this reprogramming would occur early on in tumorigenesis, caused by a lack of perfusion in the core of the tumor which would cause many of the environmental perturbations that induce the epigenetic alterations described in this paper. What is certain, is that the spatial and temporal aspects of these epigenetic modifications would be vitally important for directing tumor progression. More studies need to be completed in order to elucidate how these epigenetic changes occur directly in relation to tumor growth in models and in the patient.

## Author Contributions

MD and RG developed the idea. All authors wrote the manuscript.

### Conflict of Interest

The authors declare that the research was conducted in the absence of any commercial or financial relationships that could be construed as a potential conflict of interest.
